# Agricultural land is the main source of stream sediments after conversion of an African montane forest

**DOI:** 10.1038/s41598-020-71924-9

**Published:** 2020-09-09

**Authors:** Jaqueline Stenfert Kroese, Pedro V. G. Batista, Suzanne R. Jacobs, Lutz Breuer, John N. Quinton, Mariana C. Rufino

**Affiliations:** 1 Lancaster Environment Centre, Lancaster University, Lancaster, England UK; 2 Centre for International Forestry Research (CIFOR), Nairobi, Kenya; 3 Department of Environmental Sciences, University of Basel, Basel, Switzerland; 4 Institute for Landscape Ecology and Resources Management (ILR), Justus Liebig University Giessen, Giessen, Germany; 5 Centre for International Development and Environmental Research (ZEU), Justus Liebig University Giessen, Giessen, Germany

**Keywords:** Environmental sciences, Environmental impact

## Abstract

In many parts of Africa, soil erosion is an important problem, which is evident from high sediment yields in tropical montane streams. Previous studies in Kenya pointed to a large contribution from catchments cultivated by smallholder farmers. This led to the hypothesis that unpaved tracks and gullies are the main sediment sources in smallholder agriculture catchments of the highlands of Kenya. The aim of this study was to investigate the sediment sources with sediment fingerprinting to generate the knowledge base to improve land management and to reduce sediment yields. Four main sediment sources (agricultural land, unpaved tracks, gullies and channel banks) and suspended sediments were analysed for biogeochemical elements as potential tracers. To apportion the catchments target sediment to different sources, we applied the MixSIAR un-mixing modelling under a Bayesian framework. Surprisingly, the fingerprinting analysis showed that agricultural land accounted for 75% (95% confidence interval 63–86%) of the total sediment. Channel banks contributed 21% (8–32%), while the smallest contributions to sediment were generated by the unpaved tracks and gullies with 3% (0–12%) and 1% (0–4%), respectively. Erosion management strategies should target agricultural lands with an emphasis on disconnecting unpaved tracks form hillslope source areas to reduce sediment yields to Lake Victoria.

## Introduction

Montane headwater catchments are susceptible to soil erosion due to their topographic position on generally steep hillslopes^1–3^. Soil erosion is accelerated on land where erosive rain falls on landscapes deforested and converted to agriculture^2,4,5^. This phenomenon is evident in many places in Sub-Saharan Africa in general and in East-Africa in particular^6^. Soil particles are eroded and transported to downhill areas. The loss of topsoil reduces not only the arable soil depth, but also the content of soil organic matter, nutrients, and trace elements (e.g. organic carbon, nitrogen, phosphorus, magnesium, potassium)^7,8^, hampering agricultural productivity^7,9,10^. Element concentrations are generally enriched in the fine particle fraction (particle size < 63 µm)^8,11,12^, which are easily transported through water erosion^7^. In addition to these on-site effects, soil erosion degrades waterways as suspended sediments reduce the physical, biological and chemical water quality of streams^13,14^. These off-site effects increase adversely water treatment costs^15^, and cause the siltation of water reservoirs, which can affect water supply and hydropower generation through reduced water storage capacity^16–18^. Furthermore, sediments can be contaminated with heavy metals, nutrients and pesticides, degrading water quality (drinking water), affecting primary production and damaging aquatic habitats^8,13,19^. Agricultural intensification together with poor land management practices accelerate soil erosion^10^ and increase the number of source areas that contribute sediment to the stream network. This highlights the importance of identifying sediment sources, so that efficient management strategies can be implemented to reduce soil erosion and sediment delivery to the streams, thus reducing on- and off-site impacts.

Sediment source fingerprinting is a well-established, valuable technique to identify and apportion target sediments, in this case stream sediments, to their different sources within a catchment^20–23^. Fingerprinting begins with the classification of potential sediment sources based on reconnaissance sampling with the help of, for example, Google Earth imagery^24^ and sampling of target and sediment sources across hydrographs^21,25^. Then, potential tracer properties are selected, followed by statistical tracer selection^26–29^. Several fingerprinting studies have used different sediment properties to successfully determine their provenance. For example, Singh, Collins et al*.* and Hardy et al*.*^30–32^ used geochemical elements, while Fox and Papanicolaou, Evrard et al*.* and Mckinley et al*.*^21,33,34^ used biogeochemical properties. Mukundan et al., Owens et al. and Evrard et al.^35–37^ included fallout radionuclides and others used a combination of different fingerprinting properties^38^. Fundamental for the selection of trace elements for fingerprinting is that the tracers are present in measurable concentrations, behave in a conservative way through the mixing process (i.e. no change from source to sink) and are representative for the source^29,39,40^.

The advantage of using geochemical elements as tracers is that they provide a useful and inexpensive tool to determine rapidly a substantial number of potential tracer properties^29^. Total nitrogen and total carbon are proven to be good tracers in discriminating between surface soil erosion (e.g. topsoil of agricultural land) and subsoil erosion processes (e.g. unpaved tracks, gullies or channel banks)^41^. As the target sediment originates from upstream hillslope areas, the biogeochemical or geochemical elemental composition in the sediment source should be similar to the mixed sediment at the catchments outlet^23^. Having a large pool of tracers increases the chance to select statistically the optimum tracer composite to differentiate the target sediment to its originating sources and to quantify their relative contributions to the sediment in the stream water^29^.

A wide variety of Frequentist or Bayesian modelling approaches have been applied in un-mixing modelling^28,29,42^. The effectiveness of Bayesian sediment fingerprinting models with the use of geochemical fingerprint compositions has been demonstrated by Koiter et al*.*, Cooper et al*.* and Blake et al*.*^43–45^ and has been positively evaluated by Davies et al*.*^20^. Bayesian models apply probability distributions and incorporate prior knowledge. This leads gradually to increased knowledge from one experiment to the next and to strengthening model performance. The prior knowledge of an uncertain quantity is described by the probability models^20,40^.

The Sondu River Basin in Kenya, one of the headwaters of Lake Victoria, has experienced land use changes over the last four decades, where 25% of the largest remaining tropical montane forest in Kenya, the Mau Forest Complex, has been converted to commercial (tea and tree plantations) and smallholder agriculture^46^. A 4-year sediment monitoring study, revealed that smallholder agriculture generates a five times higher annual sediment yield than a montane forest ecosystem (South-West Mau Forest part of Mau Forest Complex) and almost three times higher than commercial agriculture (tea and tree plantations), where erosion management strategies are implemented^47^. The smallholder agriculture catchment, located in the montane headwater of the Sondu River Basin plays an important role in sediment delivery to the downstream reaches of Lake Victoria. Intensive land use practices without soil conservation techniques can increase sediment yields within the Lake basin, especially in regions with a high catchment connectivity^48^. A dense network of unpaved tracks connects hillslope areas with the stream network within the smallholder agriculture catchment and it has been observed delivering sediment-rich water to the streams during rain storms. This, together with a number of sediment studies in the tropics highlighting the importance of unpaved tracks in acting as natural drainages and discharging sediment direct to the stream network^49–51^, suggest that these tracks may be a key sediment source.

The overall aim of the study was to apportion the relative contributions of four potential sediment sources: agricultural land, gullies, unpaved tracks and channel banks to suspended sediment yields within a smallholder agriculture catchment in the headwater of the Sondu River Basin. Identifying the major sediment source is critical to develop targeted soil conservation strategies to reduce erosion, to disconnect source areas from the stream network and to decrease sediment delivery to Lake Victoria.

The main objective was to determine the main sediment source within a smallholder agriculture catchment, through (a) identification of the best sediment tracer composite of a large pool of biogeochemical and geochemical elemental properties for sediment provenance determination, and (b) estimation of the relative sediment contribution from agricultural land, gullies, unpaved tracks and channel banks using a Bayesian multivariate un-mixing model.

## Results

### Tracer selection and their discriminative behaviour

**Figure 1:**
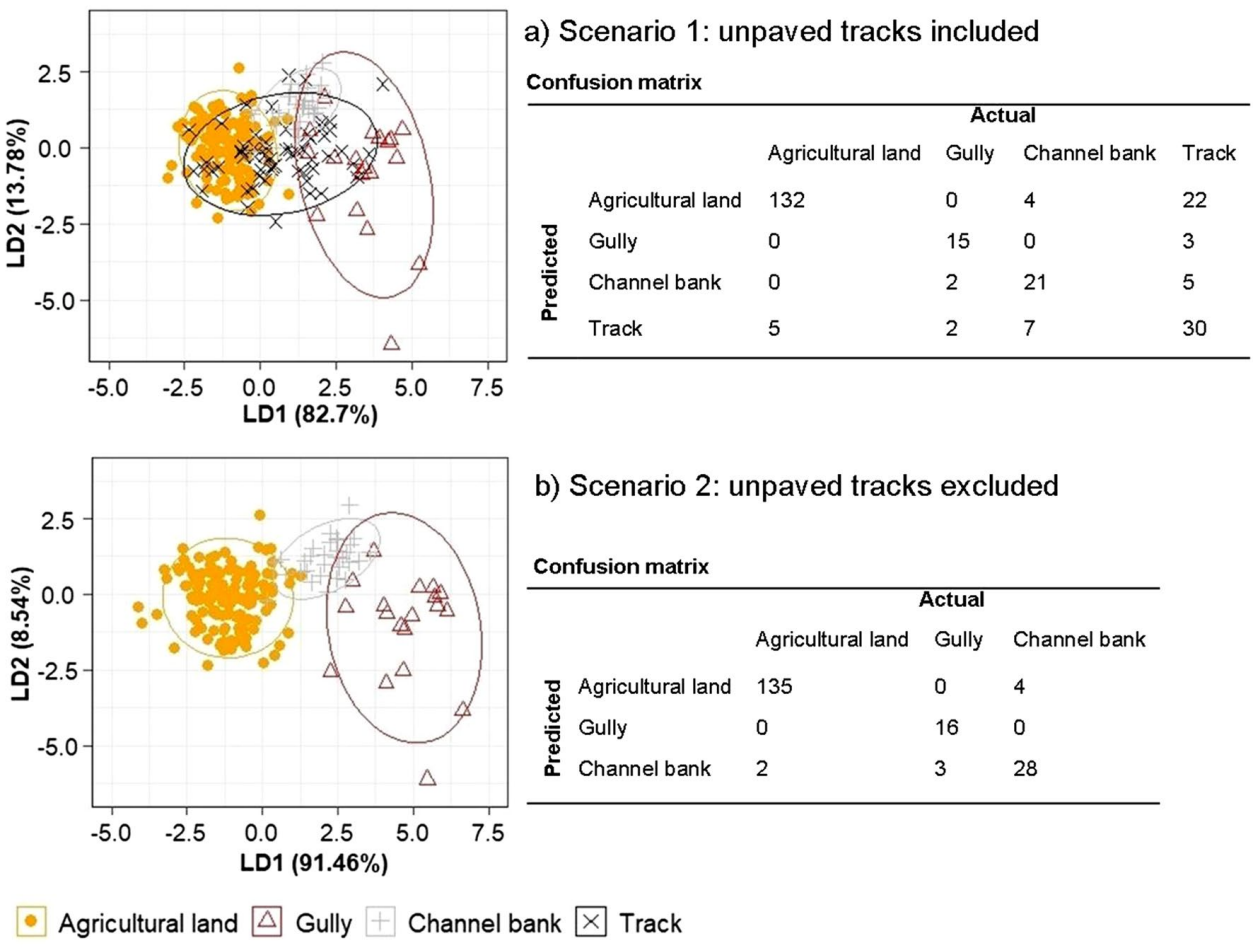
Linear discriminant analysis (LDA) for (**a**) scenario 1: unpaved tracks included & (**b**) scenario 2: unpaved tracks excluded showing the first and second discriminant functions (LD1 and LD2) of source reclassification using the selection of the composite fingerprints. Ellipses represent the 95% confidence interval. The confusion matrix shows predicted (rows) and actual (column) number of samples for each scenario.

Prior to the statistical procedure of tracer selection, P_2_O_5_ was removed as a potential tracer. Phosphorus concentrations may be highly variable in space due to inorganic fertilizer applications and because phosphorus is prone to transformation. This non-conservative behaviour may influence the fingerprinting modeling results^29,52^. Highly P_2_O_5_-enriched sediment sources were observed on samples originating from tea fields within the catchment. Of 23 geochemical and biogeochemical elements measured in each source and target sediment sample, eight (including P_2_O_5_) were removed due to values below the detection limit (with the exception of P_2_O_5_) (Supplementary Table S1). During the tracer screening, with the tracks included as a source (scenario 1), another five elements were removed because these did not comply with the range test (see “Methods” section, step 1). The ten elements remaining that passed the range test were further assessed. Rb and Zr were excluded in step 2 due to their low discrimination power to differentiate between surface and subsurface sources. A final set of eight tracers remained, comprising: TN, Al_2_O_3_, Fe_2_O_3_, K_2_O, MgO, Mn_2_O_3_, Sr and Nb. All selected composite fingerprints were able to reclassify correctly 80% of the samples in their source group after the LDA cross-validation. Because a reclassification coefficient of 80% (Table 1, scenario 1) is considered to be weak and because of the overlapping of tracers on the tracks with the other three sediment sources (Fig. 1a), we run a second scenario where tracks as individual source were excluded. The tracers remained the same for a reduced number of sediment sources and the exclusion of the tracks increased the discriminatory power of the composite fingerprints to 95% (Table 1, scenario 2).

**Table 1:** Tracers for two selection steps and reclassification coefficient (%) for scenario 1 with unpaved tracks and scenario 2 without unpaved tracks.

Scenarios	Selection step	Selected tracers	% of correctly classified samples
Scenario 1: unpaved tracks included	Step 1	TN, Al_2_O_3_, Fe_2_O_3_, K_2_O, MgO, Mn_2_O_3,_ Sr, Rb, Nb, Zr	
	Step 2	TN, Al_2_O_3_, Fe_2_O_3_, K_2_O, MgO, Mn_2_O_3,_ Sr, Nb	80
Scenario 2: unpaved tracks excluded	Step 1	TN, Al_2_O_3_, Fe_2_O_3_, K_2_O, MgO, Mn_2_O_3,_ Sr, Rb, Nb, Zr	
	Step 2	TN, Al_2_O_3_, Fe_2_O_3_, K_2_O, MgO, Mn_2_O_3,_ Sr, Nb	95

The tracers discriminated agricultural land, gullies and channel banks sediment sources well as shown in the LDA bi-plot using the first and second discriminant functions (LD1 and LD2) (Fig. 1a—scenario 1). Agricultural land and gullies do not overlap much unlike the tracer signature for the tracks that was not well discriminated. The confusion matrix shows the performance of the discriminant analysis and predicts the number of overlying samples with the tracks: agricultural land has 22 samples, the channel banks 5 and the gullies source has 3, which is also depicted in the LDA plot with highly distributed points of overlying sources (Fig. 1a). When the tracks source is removed from the analysis in scenario 2, the LDA bi-plot shows improved source discrimination for the agricultural land, gullies and channel banks sources (Fig. 1b).

### Source and target tracer composition

**Figure 2:**
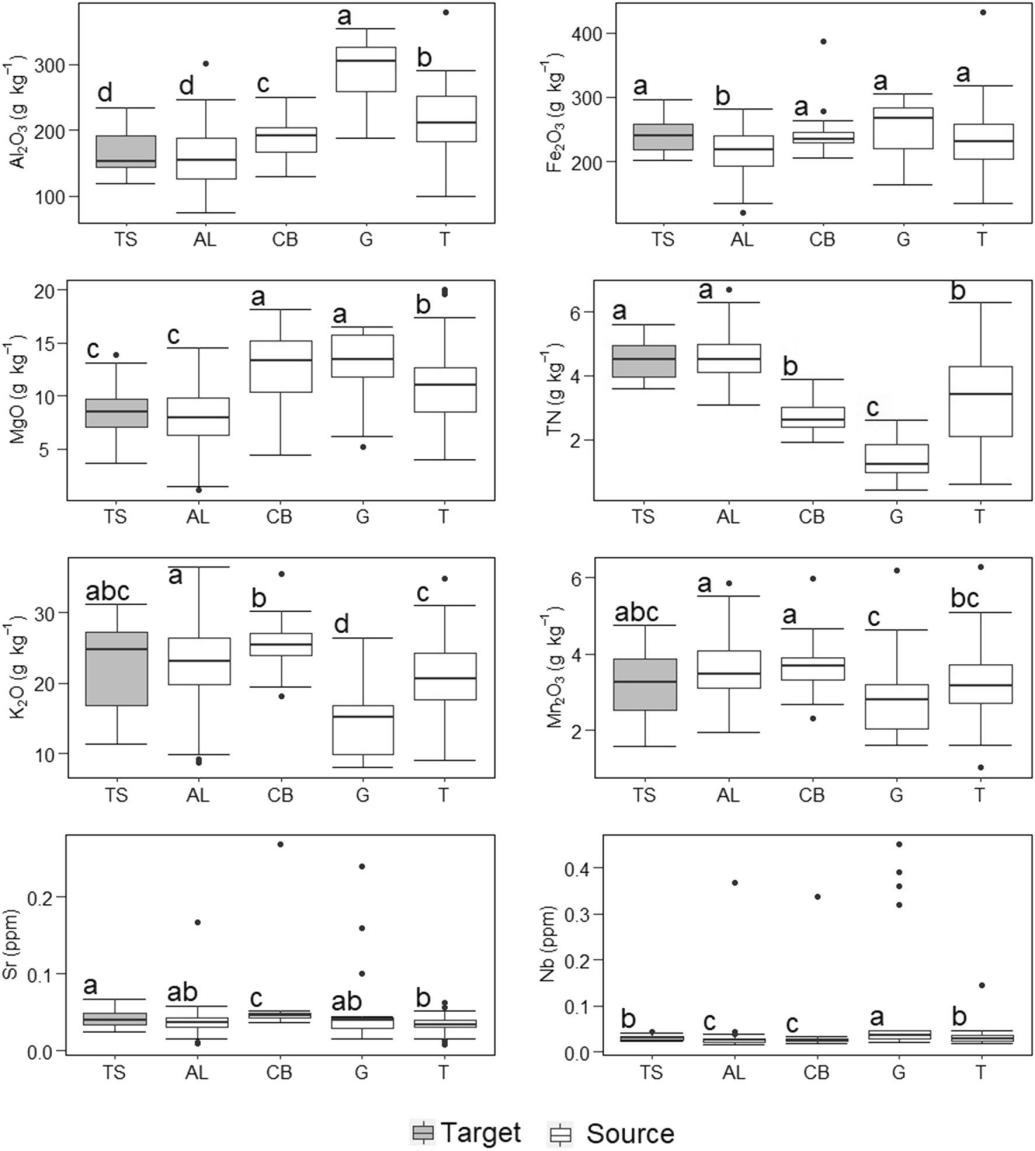
Tracer concentrations (g kg^−1^) on sediment sources (*AL *agricultural land, *CB* channel banks, *G* gullies and *T* tracks) and *TS* target sediments (letters indicate significant difference *p* < 0.05).

TN was a powerful tracer to discriminate between topsoil (agricultural land) and subsoil sediment sources (gullies, channel banks). The high TN (4.5 ± 0.6 g kg^−1^) content on the target sediment suggests that most of this sediment originated from the N-enriched agricultural lands (4.5 ± 0.7 g kg^−1^), where the sources from subsurface soil were depleted. The mean concentrations of Al_2_O_3_, Fe_2_O_3_ and MgO in agricultural land were significantly lower than in the gullies, channel banks and tracks sediment sources (*p* < 0.05). Low concentrations of these tracers at the agricultural land source corresponded with low concentrations at the target, with no significant difference between Al_2_O_3_ and MgO. The highest concentrations for Al_2_O_3_, Fe_2_O_3_ and MgO were measured in the gullies with 292.5 ± 49.6, 251.1 ± 44.6 and 12.8 ± 3.3 g kg^−1^, respectively. The mean concentrations of the tracers from the gullies were significantly lower and higher than those in the soil of agricultural land, except in Sr (*p* < 0.05). The mean concentrations of the tracks were within the range of those of the agricultural land (Fig. 2) and were not analysed further because they cannot be distinguished from the agricultural land.

### Sediment apportionment to their sources

**Figure 3:**
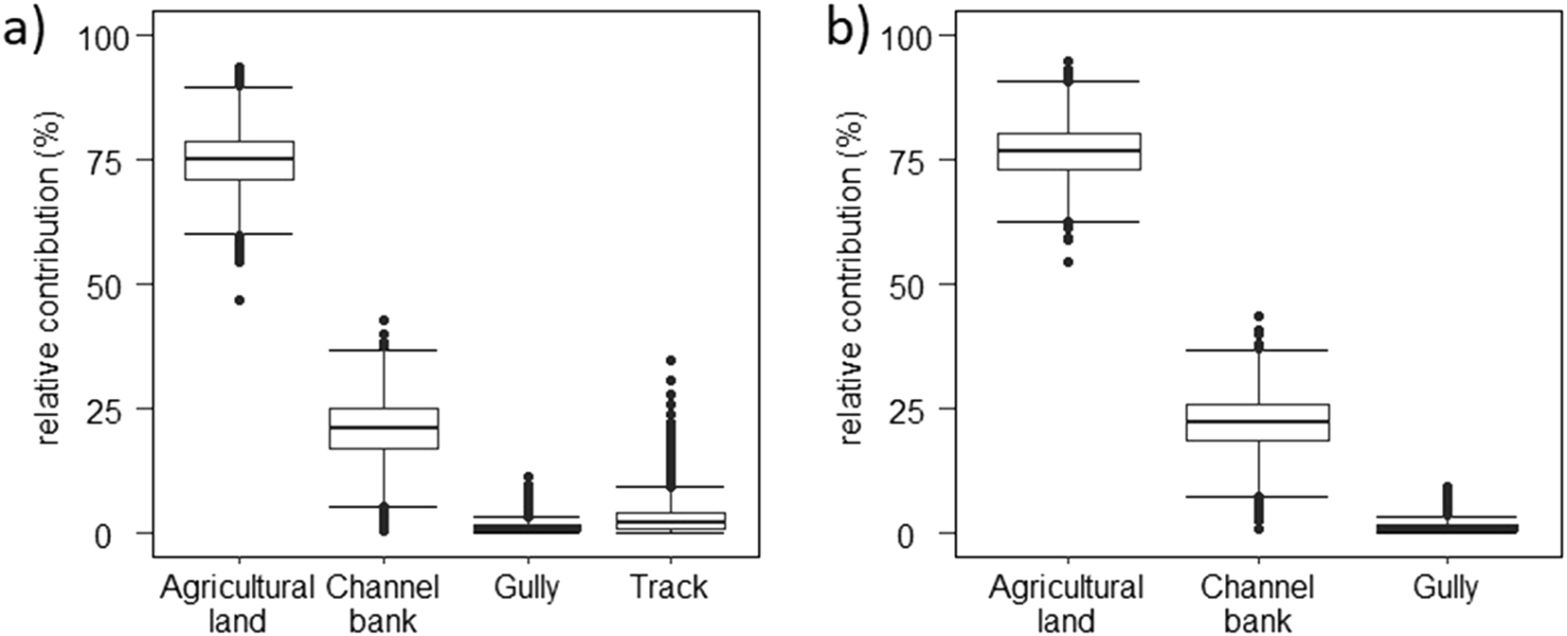
Relative apportionments of agricultural land, channel banks, tracks and gullies sediment sources based on 3,000 MCMC runs with (**a**) tracks included as a sediment source and (**b**) excluded tracks.

The un-mixing model using the selected eight tracer fingerprints on the sediment sources passed the Gelman–Rubin convergence diagnostic with a long MCMC chain run length. All the potential scale reduction factor values were < 1.05, indicating that the chain length of the MCMC was long enough. The fingerprinting results estimate the contributions of the four sediment sources to the target sediment with uncertainty estimated through the MCMC simulation procedure with 3,000 posterior realizations, which are expressed as mean and 95% confidence intervals (CI). The fingerprinting analysis shows that agricultural land accounted for 75% (95% CI 63–86%) of the target sediment at the outlet of the smallholder agriculture catchment while the channel banks contributed with 21% (95% CI 8–32%). The lowest contributions to sediment were generated by the tracks and gullies with 3% (95% CI 0–12%) and 1% (95 CI 0–4%), respectively. With the removal of tracks as independent sediment source, the relative contributions increased slightly for the agricultural land to 77% (95% CI 67–87%) and for the channel banks to 22% (95% CI 11–33%). The apportionment for gullies remained the same at 3% (Fig. 3).

### Sediment yield of each sediment source

We calculated absolute sediment generated by each sediment source by using the contributions of each source and the sediment yield of 106 t km^−2^ year^−1^ calculated at the catchment outlet by Stenfert Kroese et al*.*^47^. The contribution of agricultural land was estimated to be 80 t km^−2^ year^−1^, 22 t km^−2^ year^−1^ by channel banks, 3 t km^−2^ year^−1^ by tracks and 1 t km^−2^ year^−1^ by gullies. In addition to the absolute sediment contributions per catchment area (27 km^2^), we evaluated the sediment yield of each specific sediment source per unit area within the catchment. The total annual sediment load (2,892 t) was multiplied by the relative sediment contribution to obtain the sediment yield per source area (Table 2). This was then divided by the area occupied by the different sources to calculate the sediment yield per unit area, i.e. the amount of sediment originating from one square kilometre of land covered by that particular sediment source. By including the unit area, the sediment yield was highest for channel banks (6,073 t km^−2^ year^−1^), followed by gullies (567 t km^−2^ year^−1^), tracks (150 t km^−2^ year^−1^) and the lowest for agricultural land (85 t km^−2^ year^−1^).

**Table 2:** Relative (%) and absolute sediment contributions (t km^−2^ year^−1^) weighted per source area and catchment area for tracks included and excluded of the sediment apportionment within a smallholder agriculture catchment in the Sondu Basin of Kenya.

Scenario	Sources	Surface area km^2^	Relative contribution %	Absolute contribution per source area t km^−2^ year^−1^	Absolute contribution per catchment area t km^−2^ year^−1^
Scenario 1 (tracks included)	Agricultural land	25.7	75 (63–86)	85 (71–97)	80 (67–91)
	Gullies	0.1	1 (0–4)	567 (0–2,268)	1 (0–4)
	Channel banks	0.1	21 (8–32)	6,073 (2,314–9,254)	22 (8–34)
	Tracks	0.6	3 (0–12)	150 (0–598)	3 (0–13)
Scenario 2 (tracks excluded)	Agricultural land	26.8	77 (67–87)	83 (72–94)	82 (71–92)
	Gullies	0.1	1 (0–4)	567 (0–2,268)	1 (0–4)
	Channel banks	0.1	22 (11–33)	6,362 (3,181–9,544)	23 (12–35)

In brackets 95% confidence interval.

## Discussion

The tracer composite of scenario 2, excluding tracks, explained 95% of classified sources, which is considered to be robust^53^. The selected tracers showed clear differences in their concentrations between sources, caused by underlying biogeochemical and pedological processes related to each element. TN is an important plant nutrient and therefore a powerful tracer to discriminate between surface (agricultural land) and subsurface (gullies and channel banks) sediment sources. The higher concentrations at the soil surface reflect the inputs from atmospheric sources, fertilization, animal manure and biological N fixation^54,55^. This decrease in TN concentrations with depth helped differentiate between surface and subsoil sources in this study, similarly to previous fingerprinting studies e.g. Russel et al*.*, Gellis et al*.* and Collins et al*.*^56–58^. To characterise the natural variability in the distribution of TN among sediment sources, we collected a larger number of source samples than typically obtained as recommended by Laceby et al*.*^59^ As we are sampling suspended sediment derived during high flow events when sediment travel times are short, the reduction in TN concentrations due to biological processes, such as mineralization are expected to be small^60^.

Geochemical tracers also showed differences between surface and subsoil sources resulting from weathering and pedogenic processes. For example, Al_2_O_3_, Fe_2_O_3_ and MgO were enriched in subsoils and depleted in topsoils, whereas the opposite was observed for K_2_O and Mn_2_O_3_. This elemental behaviour was observed in a fingerprinting study by Tiecher et al*.*^61^, where aluminium was a strong tracer to differentiate subsoil and surface sources in Southern Brazil. Clay content is expected to increase with soil depth with enriched residual Al_2_O_3_ and Fe_2_O_3_ concentrations^62,63^. The subsoil is expected to be enriched with trace elements (Sr and Nb), because clay acts as a sink for these elements^64^. Tropical climates with high annual rainfall intensify weathering processes of mollic Andosols and humic Nitisols. Less weathered subsoil may therefore exhibit higher concentrations in Al_2_O_3_, Fe_2_O_3_ and MgO^65,66^. The elemental composition of the topsoil may not have been as influenced by pedological processes as deeper horizons which are closer to the parent material^66^. Igneous rocks are rich in Fe_2_O_3_ and MgO^67^, thus providing a good discriminator for subsoil sources with higher concentrations of these elements. Conversely, K_2_O and Mn_2_O_3_ are plant macro- and micronutrients and are recycled by plants mainly in the topsoil. Vegetation on agricultural land may transport these heavier elements (K_2_O and Mn_2_O_3_) to the topsoil, a process that does not occur in gullies or unpaved tracks which are mostly bare^55,68^.

Although the tracers helped to differentiate between surface and subsoil sources, they could not differentiate clearly between the different land uses, such as grasslands and cropping fields. Only 67% of the sources were correctly classified by the tracers, which is why the different land uses were categorized into one sediment source group called agricultural land (Supplementary Figure S1).

The results of this study show that agricultural land is the main sediment source in the smallholder agriculture catchment with a contribution of 77%. This is reflected in the chemical composition of the target sediments, with tracer concentrations similar to those expected in agricultural topsoils (Fig. 2). A similar source apportionment was observed in two studies in steep cultivated catchments in South-Brazil, where surface erosion originates mainly from agricultural land with smaller to minor contributions from channel banks and unpaved tracks^50,61^. Also in a Zambian catchment, sediments were found to mainly originate from communal land cultivation, which covered about 70% of the catchment area. In that study, a dense network of trackways was thought to connect overgrazed hillslope areas with the stream network delivering sediment from the bush grazing sediment source^69^. These findings further elucidate the need for soil conservation strategies in other tropical catchments with a current trend towards agricultural expansion^70^.

In our study, unpaved and highly compacted tracks often run parallel to the slope, thus acting as conduits during rainfall events and turning into ephemeral streams carrying surface runoff with high loads to the streams. Due to their frequent use and position, we hypothesised that these tracks would be the main sediment source, as suggested by other sediment fingerprinting studies^71,72^. However, we found tracks to be only a minor contributor to the overall catchment sediment budget. Their role as conduits from the surrounding source areas to the stream may explain the overlap of tracers in the track samples with the remaining sources. Besides, unpaved tracks may have been converted from agricultural land which might also lead to similarity in their tracer characteristics. Consequently, unpaved tracks were excluded as an independent sediment source, because the tracer composite could not clearly distinguish from agricultural land, leading to 80% of correctly classified samples.

Although the sediment yield per unit area of channel banks, gullies and unpaved tracks are large, they are not the main contributors to sediments at catchment scale. However, the sediment contribution increases at a smaller scale, which was also observed by Tiecher et al*.*^61^ for sub-catchments < 3 km^2^. The scale-dependency points to one main finding: that sediment loss from agricultural land per unit area is relatively low, but because it occupies most of the catchment area, it contributes the greatest proportion of the sediment yield. Channel banks, gullies and unpaved tracks are significant local hot spot sources, but their overall contribution is of lower significance. Instead, gullies and unpaved tracks act as significant conduits, connecting hillslopes with the streams. When weighting sediment contributions to the whole catchment, a dilution effect by the catchment area decreases the sediment supply from the scale-dependent source areas. When scaling up to the Sondu River Basin, the large proportion of agricultural areas within the whole catchment raises a concern, as it discharges an increasing amount of sediment at the outlet into Lake Victoria. However, source apportionment might vary depending on sampling locations of target sediment in nested sub-catchments due to catchment-scale dependent sediment dynamics^45^. A large scale campaign may give additional information of changing source contributions within the whole catchment.

An increased sediment yield in the streams originating from nutrient-rich topsoil of agricultural land contributes to eutrophication of waterbodies^73^ and threatens crop yields^9,10^. The loss in annual agricultural productivity will decrease farmer’s revenue, but, more importantly, it will threaten food production and security for future generations due to slow rates of soil formation^74^. This is a further impediment for the already low agricultural productivity of the country, where soil erosion is a primary constraint to improving yields^75^. Cohen et al*.*^76^ estimated the magnitude of soil erosion losses for Kenya’s economy to be US$ 390 million annually or 3.8% of the gross domestic product. In addition to the on-site impacts, the nutrient rich topsoil ending up in the streams may lead to an oversupply of macronutrients (N and P)^8,13^ resulting in an eutrophic state of watercourses, turning rivers from natural to degraded^77^. The discharge of nutrient rich sediments from the montane streams to the outlet of the Sondu River Basin is in particular a threat for the already enriched Lake Victoria^78^ and degrades the lake’s water quality. This emphasizes the need for targeted mitigation measures to decrease hidden costs of on- and off-site impacts of soil erosion.

Since the majority of the annual sediment yield in the study area originates from agricultural land, this should be the main target for soil management strategies. The largest proportion of the total sediment budget (59%) is generated during the start of the long rainy season, which coincides with the start of the planting season (March–April), large areas of bare soil or low ground vegetation cover on agricultural land^47^. Vegetative buffer strips alternated with fields cultivated with perennial crops can reduce the pace of surface runoff and can trap eroded material. This practice could lead to a significant reduction in soil loss from cropping fields, especially during the planting season^79^. Buffer strips could also act as source of livestock feed for example to cultivate fodder crops such as Napier grass (*Pennisetum purpureum*). Currently, erosion buffer strips are only occasionally observed on agricultural land within the catchment. Our sampling campaign was restricted to one hydrological period (April–June 2019), which limits the characterization of seasonal dynamics of sediment sources. In the future, target sediment could be sampled throughout different seasons (short and long rainy season and dry season) to test whether there are temporal changes in source contributions.

A small floodplain in a steep, narrow valley floor provides limited space for sediment storage in the study area. This increases the need for enhanced on-field erosion mitigation measures to protect soil on hillslopes. Other methods to reduce soil erosion from agricultural land include terracing and the cultivation of cover crops, which can boost crop productivity. Cover crops can protect the surface from erosive rainfall by reducing the energy of raindrops^7,80^, as well as enhancing soil organic matter^81^. Terracing on steep slopes, especially in the study area, could reduce surface erosion through decreasing slope length and steepness in dividing the slope into smaller segments^10^. In Kenya, *fanya juu* terracing is commonly used on moderately steep slopes up-to 20%^82^. The use of these terraces on croplands have increased crop yields by 25% in East-Africa^10^ and help the recovery of soil organic matter^9^ due to reduced soil erosion.

Although the unpaved tracks are not a main source of sediment, according to our study, they show high erosion rates per area, acting as hot spots, and therefore effective management strategies should still be targeted at unpaved tracks. Other studies have emphasized unpaved roads as landscape features with high erosion rates and as significant contributors of sediment to the stream network^83–85^. In Malaysia erosion rates were estimated up to 320 ± 24 t ha^−1^ year^−1^ originating from steep skid trails (> 20% gradient) in logged forests^84^, while in South-Brazil the need for erosion control programmes were stressed onto unpaved tracks as they form into perennial landscape features^61,86^. The potential sediment contribution could increase with the length of a track, where the gullied track length defines the distance of sediment transport^85^. These results stress on the importance of disconnecting rural unpaved tracks from the drainage system to lower the contribution of sediment from surrounding hillslopes in tropical disturbed catchments. Sediment yield could be further reduced by disconnecting cultivated hillslope areas in the smallholder catchment from the highly incised and mainly long unpaved tracks to reduce sediment delivery during storm events. In addition, water pathways on the tracks could be diverted to adjacent agricultural lands to attenuate the routing of surface runoff by unpaved tracks to the streams.

Riparian zones, as described within Kenya’s Water Act, are defined as a buffer of 30 m along the watercourse^87^. Riparian vegetation is efficient in buffering significantly surface runoff, reducing suspended sediments and nutrient discharge from agricultural land^88–90^, but also prevents the collapse of channel banks^91^. A dense riparian vegetation can strengthen channel bank stability through a dense rooting system^92,93^. Tiecher et al*.*^61^ showed that the presence of an intact riparian forest disconnected cultivated areas from the stream network, decreasing the sediment contribution from cropland sources compared to areas with a degraded riparian forest in a catchment in Southern Brazil. The same was found in the Chesapeake Bay watershed, USA, where a forested floodplain increased the amount in trapped sediment^57^. In our study area, widespread cultivation within the buffer zone and the absence of riparian vegetation may result in the higher transfer from soil particles originating from hillslopes areas. Furthermore, increased pressure on available arable land in the catchment is leading to conversion from wetlands to agricultural lands. With this conversion, another important sediment trap is lost^94,95^. This emphasises the need for appropriate management of riparian areas to buffer hillslope sediments and to strengthen channel banks.

In our study area, gullies provided the smallest contribution (1%) to the sediment budget. Although the overall sediment contribution of channel banks and gullies remained low, both sources are potential soil erosion hot spots at the local scale. Furthermore, the absence of management strategies might increase the relative sediment contribution with proceeding gully head or wall erosion^85^. Gully rehabilitation could be achieved by replanting trees and increasing vegetation cover. Further gully expansion could be impeded in diverting waterways to avoid lateral runoff water entering through gully walls and keeping away livestock and human movement from highly eroded areas^10^.

## Conclusions

We aimed to identify the relative contribution of four sediment sources: agricultural lands, unpaved tracks, gullies and channel banks using un-mixing modelling (MixSIAR) to assess the relative contribution to suspended sediment at the outlet of a smallholder agriculture catchment in the highlands of Kenya. Due to the topography of this montane headwater catchment, there is a strong catchment connectivity between hillslope areas and the stream, with limited deposition of sediment in the narrow floodplain with a steep valley floor. The sediment fingerprinting demonstrated that topsoil of agricultural lands (77% with 95% CI of 67–87%) is the main source of suspended sediment. A dense network of unpaved trackways fragments the landscape of the catchment and acts as conduit between hillslope areas and waterways. However, these tracks could not be identified as individual sediment source. The lack of an intact riparian vegetation results in subsoil channel bank erosion as a second major sediment contributor with 22% (11–33% 95% CI), whereas gullies contribute only a small proportion with 1% (0–4% CI-95) to the sediment yield.

Sediment fingerprinting may be applied in other tropical agricultural catchments to further develop the knowledge base of sediment source areas where problems of soil erosion and the accumulation of sediments are a concern. Based on our findings, we speculate that in catchments dominated by smallholder agriculture in large parts of East-Africa in combination with poor road network and lack of appropriate runoff and sediment management strategies agricultural land is the main source of sediment. These results emphasize the need for targeted erosion mitigation strategies on agricultural land to limit soil erosion and control annual sediment yield. Moreover, the large proportion in agricultural areas in the highly populated highlands of Kenya raises concern in discharging an increasing amount of sediment to the streams, consequently, impacting further the water quality of Lake Victoria.

## Methods

### Catchment description

**Figure 4:**
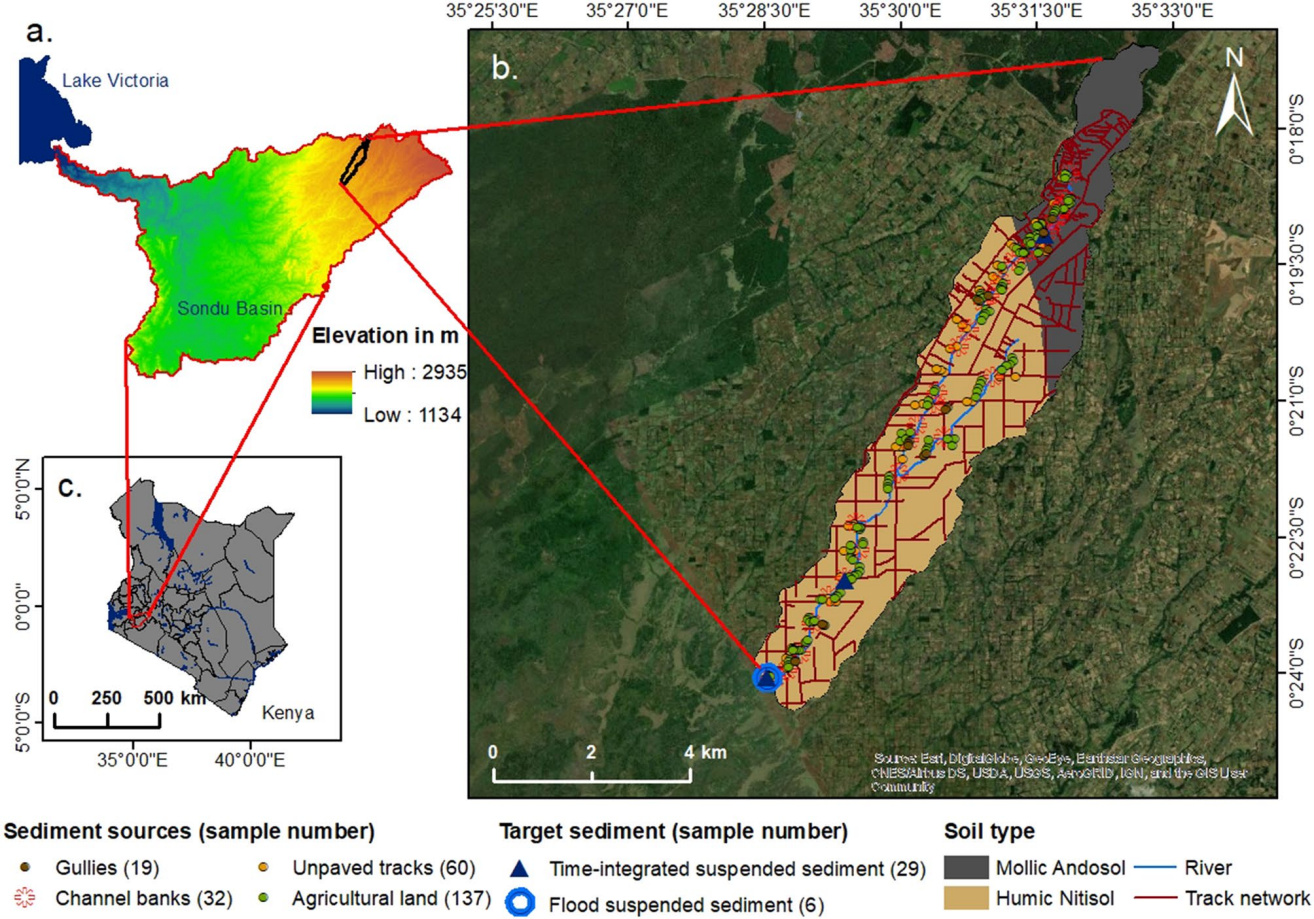
(**a**) Elevation map (SRTM digital elevation model 30 m resolution^96^) of the Sondu River Basin with outlet to Lake Victoria and (**b**) pedological map with source and target sediment sampling points (Geology data from the Soil and Terrain database for Kenya (KENSOTER) version 2.0^97^ with imagery basemap^98^ of the smallholder agriculture catchment in the highlands of (**c**) Kenya (map generated using ArcMap 10.4 (10.4.1)^99^.

The study area is a smallholder agriculture catchment (27 km^2^) located in the western highlands of Kenya. It is part of the headwater of the Sondu River Basin, which drains into Lake Victoria (Fig. 4). The catchment has a dry season (January to March) and a wet season with two rainfall peaks: the long rains from March to May (77–277 mm month^−1^) and the short rains from June to September (~ 160 mm month^−1^) with continued intermitend rainfall events between seasons. The annual rainfall ranged from 1,400 to 1,800 mm (period 2015–2018)^47^.

Throughout the catchment steep slopes (maximum 52.4%) characterise the montane area with an altitude range from 2,389 m a.s.l. at the catchment outlet to 2,691 m a.s.l. at the source. The geology is characterised by lava flows of volcanic (72%) and pyroclastic (28%) parent material^97^. Phonolites, a member of a group of extrusive igneous porphyritic rocks (lavas), predominate in this area^100^. There are two soil types: mollic Andosols (28%) in the upper part of the catchment and humic Nitisols (72%) in the middle and lower part (Table 3). They are well drained, deep soils (up to 5–6 m in depth) consisting of dark-brown loamy to clayey soils^101^ with moderate to high organic matter content^102^. Nitisols are characterised by high concentrations of free iron. Mollic Andosols are formed following rapid weathering of porous volcanic material. Dominant minerals include allophanes, with hydrous aluminosilicates, and ferrihydrite. Aluminium-humus complexes protect the organic matter from bio-degradation^103^. Due to their high porosity these soils have excellent internal drainage with infiltration rates of 400 mm h^−1^ ^104^. Nevertheless, desiccated Andosols, common after deforestation, have low water permeability that makes them susceptible to water erosion^103^.

**Table 3:** Physical characteristics of a smallholder agriculture catchment in the South-West Mau, Kenya.

Area	km^2^	27.2
Outlet coordinates^a^	Longitude	35°28′31.7316″E
	Latitude	0°24′4.0248″S
	Stream order (Strahler)	1, 2
Drainage density	km km^−2^	0.64
Track density	km km^−2^	4.28
Altitude range	m a.s.l	2,389–2,691
Mean slope ± SD	%	11.6 ± 6.7
Geology^b^		Igneous rock (Volcanic) (72%)
		Pyroclastic (28%)
Dominant soils^b^		Humic Nitisol (72%)
		Mollic Andosol (28%)
Soil profile	Andosol	AC to ABC
	Nitisol	AB(t)C
Land use		Perennial & annual crops, woodlots, grazing land

*SD* standard deviation.

^a^WGS 1984 UTM Zone 36S.

^b^Geology data from the Soil and Terrain database for Kenya (KENSOTER) version 2.0.

**Figure 5:**
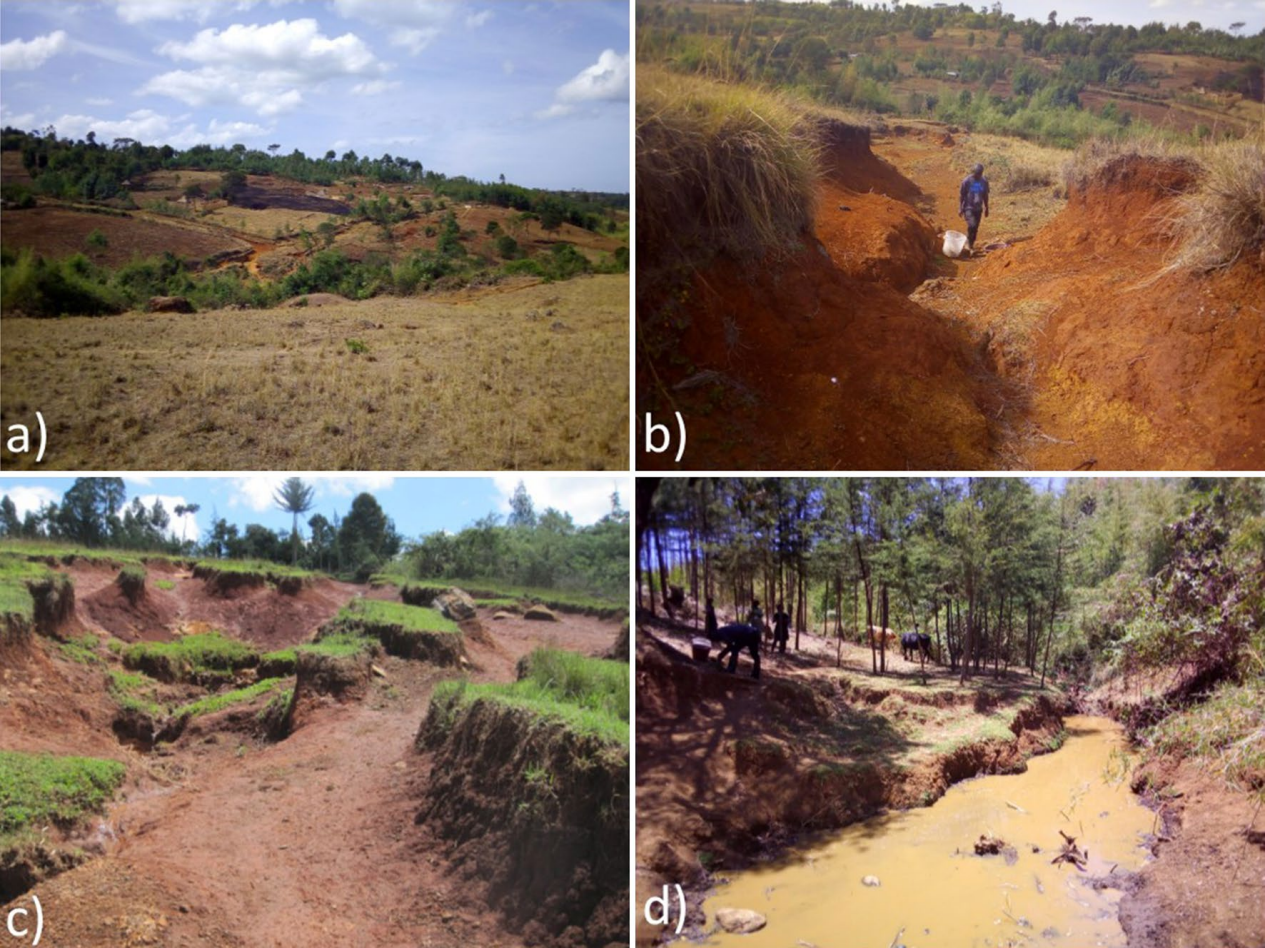
(**a**) Characteristic landscape of the smallholder agriculture catchment, (**b**) hillslope gully, (**c**) unpaved eroded track connecting with the stream and (**d**) exposed channel bank.

Historically, most of the catchment was covered by the Mau Forest Complex, which was cleared for agricultural land over the last four decades^46^. Today their fertile volcanic soils are planted with a variety of crops including maize, interspersed with beans, potatoes, millet, cabbage and tea on small farms (< 1 ha). Eucalyptus (*Eucalyptus* ssp.), cypress (*Cupressus* ssp.) and pine (*Pinus* ssp.) woodlots are interspersed with croplands and grazing land. Tillage in the form of hand-hoe cultivation and ploughing with oxen are the common practices for soil preparation. A dense network of unpaved tracks, organized in a rectangular grid, connect hillslopes with the stream network. These tracks regularly used by people and livestock often become incised to form gullies. Gullies can be found on steep slopes or around unprotected springs. Stream channel banks are susceptible to erosion due to the absence of riparian vegetation (Fig. 5). The catchment supplies on average 106 ± 46.8 t km^−2^ year^−1^ of fine suspended sediment at the outlet^47^.

### Source and target sediment sampling design

**Figure 6:**
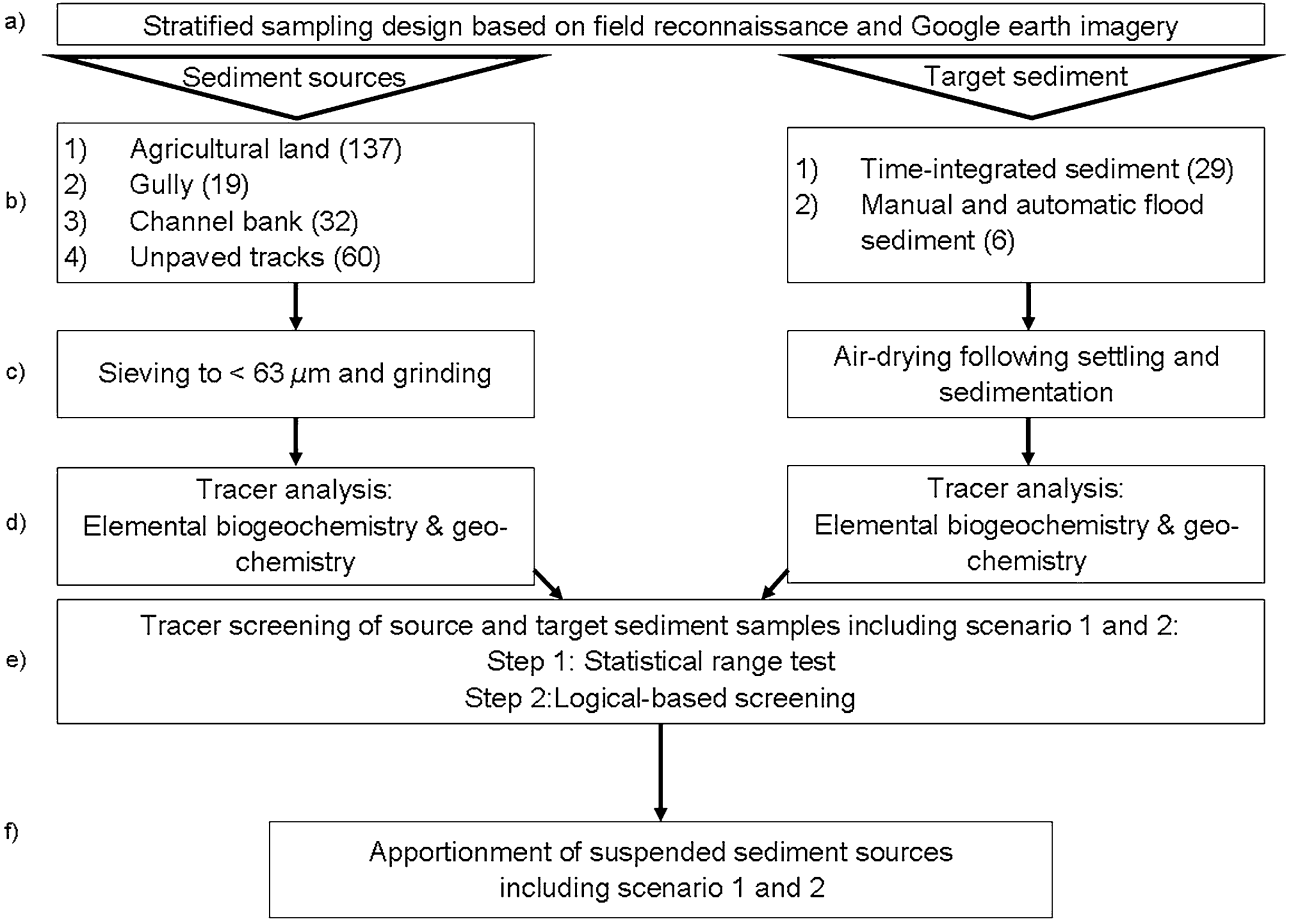
Flow diagram of the sediment fingerprinting sampling, analytical and statistical procedure (in brackets sample number).

Source and target sediment sampling were conducted from April to June 2019. A stratified sampling design was used which was based on field reconnaissance and Google earth imagery. Sampling points were restricted to areas where soil mobilization and on-field transport processes from hillslope areas were potentially connected with the stream network. To represent material susceptible to runoff detachment, sediment source samples were collected by scraping the uppermost layer of soil (~ 2 cm) of agricultural land and unpaved tracks (Fig. 5a,c). The agricultural land source group included annual cropping field and fallow land, tea plots and grassland. Grasslands were not distinguishable from the cropland sources based on similar tracer concentrations we believe because they are used in rotations with croplands (Figure S1). Each agricultural land sampling point (*n* = 137) was composed of multiple subsamples of the same land use collected along parallel transects within a radius of 25 m around a sampling point pre-selected visually on recent Google earth imagery. Multiple unpaved tracks samples were collected along transects on the track width, track length and of track walls and combined to a bulk sample (*n* = 60). Samples of gullies (*n* = 19) and channel banks (*n* = 32) (Fig. 5b,d) were collected from several points along vertical and horizontal profiles of the subsoil (up-to a depth of 2 m) and combined into a single sample. Channel bank samples were only taken on sites with exposed banks without vegetation cover. Litter or vegetated cover was removed prior to taking soil samples. Each surface and subsoil sample was composed of 10–20 subsamples. A plastic trowel was used for sample collection to avoid metal cross-contamination (Fig. 6a,b).

Target sediment samples consisted of flood sediment samples (*n* = 6) and time-integrated suspended sediment samples (*n* = 29). Flood sediment samples were collected manually and automatically at the catchment outlet. Manual event-based samples were retrieved with bulk river water samples (~ 10 L). In addition, two automatic water samplers (3,700 Full-size portable sampler, Teledyne ISCO, Lincoln, USA) were used to collect 0.5 L samples during the rising and falling limbs of the storm hydrograph. Sediment from the bulk river water samples and the automatic sampling were extracted through the settling and sedimentation method and then air-dried (Fig. 6a–c). For time-integrated samples three sediment traps following the method by Phillips et al*.*^25^ were installed at the outlet and at two locations upstream of the outlet (Fig. 4) and samples were collected every 3–5 days.

### Sediment source and target sediment processing and analysis

Sediment source samples were sieved to < 63 µm in the field to facilitate direct comparison to the target sediment samples^29,105^. Sediment source samples were air-dried and ground for further analysis (Fig. 6c). All sediment samples (sources and target) were analysed in the laboratory for their chemical properties, including total carbon (TC) and total nitrogen (TN) content^21,106^ and major and minor elemental geochemical constituents^32,106^ as potential sediment tracers. For TC and TN concentration a sub-sample of 15 mg was wrapped in tin capsules and combusted in an elemental micro-analyser (Elementar vario MICRO Cube, Elementar Analysensysteme GmbH, Langenselbold, Germany) at 950 °C. A handheld XRF spectrometer (Bruker Tracer IV-SD, Bruker, Kennewick, WA USA) that uses energy dispersive X-ray fluorescence (EDXRF) was employed to determine the following major elements: Al_2_O_3_, CaO, Fe_2_O_3_, K_2_O, MgO, Na_2_O, P_2_O_5_, SiO_2_ and TiO_2_ and trace elements: Ba, Cr, Cu, Mn_2_O_3_, Nb, Ni, Pb, Rb, Sr, Y, Zn and Zr. For this analysis, a sub-sample of 500 mg was placed onto a thin film to measure trace elements at a setting of 40 kV and 15.7 µA and major elements at an excitation of 15 kV and 35 µA under vacuum with Helium gas. For each sample the mean of three replicates was used for further analysis (Fig. 6d).

### Tracer selection procedure

To select tracers for optimal discrimination of different sources, a verification procedure is needed to prove the strength of composite tracers for source apportionment. We followed a two-step tracer selection approach based on a statistical tracer screening combined with a logical-based selection (step 2) for an optimum range of tracers (Fig. 6e). This two-step approach differs from un-mixing models under the Frequentist framework, where commonly a three-stage statistical procedure is aimed to select a minimum range of tracers^20,107^. The use of several tracers including weak tracers increases the explanatory power of un-mixing modelling under the MixSIAR Bayesian framework^53,92,108^. The covariance structure in the MixSIAR un-mixing modelling reduces redundancy and therefore a discriminant function analysis to create a composite of a minimum of tracers is not required^40^.

The range test was used to exclude elements that do not differentiate sediment sources^44^. A target element concentration should be in range with the source mixing polygon showing whether a tracer on the target sediment is enriched or depleted compared to the sediment sources^35,105^. Consequently, the range test analyses the conservative behaviour of the selected tracers^29^. Elements plotting outside the mixing polygon are removed from the subsequent analysis. Here, the mean and standard deviation of log-transformed concentrations of the target sediment should be within the ranges of the concentrations of the sediment sources. Tracers outside this criterion violate the numerical modelling assumptions and may lead to false results of the un-mixing model^109^. The remaining elements selected by the range test were screened for their discrimination power to distinguish between surface and subsurface sources. The discriminating power of the selected tracer composite was evaluated using the reclassification coefficient and linear discriminant analysis (LDA) bi-plots. Based on the evaluation of the discrimination power, two different scenarios were run: scenario 1 with the inclusion of the unpaved tracks as individual source, and scenario 2 excluding the tracks from the analysis. Tracers were screened independently for each scenario. For statistical analyses, the R software^110^ was used together with the packages MASS^111^ and klaR^112^ for the LDA cross-validation.

### Modelling source apportionment

To estimate the relative contribution of each source at catchment level, a Bayesian un-mixing model was applied using the MixSIAR model (Stable Isotope Analysis in R)^113^. The MixSIAR model runs in the JAGS software (Just Another Gibbs Software)^114^ to carry out a Markov chain Monte Carlo (MCMC) sampling together with a Bayesian analysis that produces diagnostics, density plots a posteriori and summary statistics. Prior to modelling, element concentrations were log transformed to approach normality. The MixSIAR model uses the mean and standard deviation of the tracers as inputs. The model parameters, i.e. the proportions of tracer compositions of the sediment sources, are treated as random variables. Parameter uncertainty is specified by using three different stages of probabilistic predictions by: (1) using the Dirichlet distributions to determine prior probability distributions for parameters, (2) constructing a likelihood function for the model and (3) using the Bayes rule to adjust prior distributions based on observed data to derive the posterior probability distribution^115^. The basic linear un-mixing model takes the following form:

1 $${C}_{ti}=\sum _{s=1}^{m}{P}_{s}{C}_{Si}$$

with ${\sum }_{s=1}^{m}{P}_{s}=1$ and $0\le {P}_{s}\le 1$. ${C}_{ti}$ is the concentration of tracer *i* of the target sediment *t*, ${P}_{s}$ is the proportional contribution from sediment source *s*, and ${C}_{Si}$ is the concentration of tracer *i* of the sediment source *s* with the number of sediment source *m*. The estimated discrimination of sediment sources was carried out with the MCMC sampling on three “long” chains of length 300,000 with a 200,000-sample burn-in and a jump length of 100 to minimize autocorrelation between runs, yielding 3,000 model values of proportional source contributions. Un-mixing model convergences were assessed with the Gelman–Rubin diagnostic, were the chain length was increased when > 5% of total variables was above 1.05^113^. The un-mixing modelling results of the relative contributions of each sediment source are presented as the average with 95% confidence interval for scenario 1 and 2 (Fig. 6f).

## Supplementary information

Supplementary Informations.

## Data Availability

The datasets generated and analysed during the current study are available online https://doi.org/10.17635/lancaster/researchdata/365 hosted by Lancaster University, United Kingdom.
