# Supplementary material for: Agricultural land is the main source of stream sediments after conversion of an African montane forest
Source: Sci Rep. 2020 Sep 9;10:14827. doi: 10.1038/s41598-020-71924-9 (PMC7481190; doi:10.1038/s41598-020-71924-9)
Supplement: Supplementary file 1 — Supplementary Informations. [file 41598_2020_71924_MOESM1_ESM.pdf]

Electronic supplementary material

Agricultural land is the main source of stream sediments after conversion of an African montane forest

Jaqueline Stenfert Kroese[1,2,*], Pedro Batista[3], Suzanne Jacobs[5], Lutz Breuer[4,5], John N. Quinton[1], Mariana C. Rufino[1,2]

[1]Lancaster Environment Centre, Lancaster University, Bailrigg, Lancaster, LA1 4YQ, England, UK

[2]Centre for International Forestry Research (CIFOR), Nairobi, Kenya,

[3]Department of Environmental Sciences, University of Basel, Basel, Switzerland,

[4]Institute for Landscape Ecology and Resources Management (ILR), Justus Liebig University, Giessen, Germany,

[5]Centre for International Development and Environmental Research (ZEU), Justus Liebig University, Giessen, Germany

*J.stenfertkroese@lancaster.ac.uk

Table S1 Average concentrations of TN, TC and geochemical elements of source and target sediment samples (* removed prior analysis).

	Agricultural land	Tracks	Gullies	Channel banks	Target sediment
TN [g kg^{-1}]	5	3	1	3	4
TC [g kg^{-1}]	53	41	16	29	50
Na_2O [g kg^{-1}]	32	33	30	30	34
MgO [g kg^{-1}]	8	11	13	12	9
Al_2O_3 [g kg^{-1}]	157	214	293	185	166
SiO_2 [g kg^{-1}]	478	466	454	455	449
P_2O_5 [g kg^{-1}]*	1	1	1	1	1
K_2O [g kg^{-1}]	23	21	14	25	23
TiO_2 [g kg^{-1}]	30	34	36	40	30
Mn_2O_3 [g kg^{-1}]	4	3	3	4	3
Fe_2O_3 [g kg^{-1}]	213	232	251	241	240
CaO [g kg^{-1}]	9	9	7	11	11
Ba [ppm]*	0.03	0.03	0.02	0.02	0.02
Cr [ppm]*	-0.03	-0.01	-0.01	-0.03	-0.02
Ni [ppm]*	0.00	0.00	0.00	0.00	0.00
Cu [ppm]*	0.00	0.00	0.00	0.00	0.00
Zn [ppm]*	0.01	0.01	0.01	0.00	0.00
Pb [ppm]*	0.00	0.00	0.00	0.00	0.00
Rb [ppm]	0.00	0.00	0.00	0.00	0.00
Sr [ppm]	0.04	0.03	0.05	0.05	0.04
Y [ppm]*	0.00	0.00	0.00	0.00	0.00
Zr [ppm]	0.15	0.16	0.52	0.16	0.15
Nb [ppm]	0.03	0.03	0.11	0.04	0.03

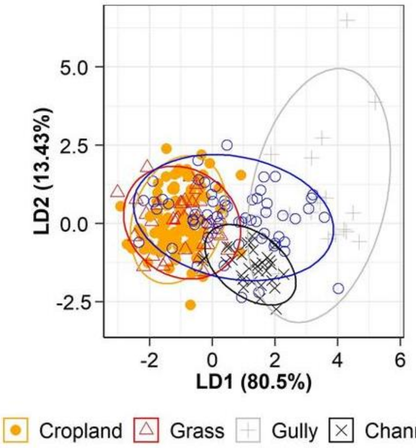

		Actual				
		Cropland	Grassland	Gully	Channel bank	Track
Predicted	Cropland	95	31	0	3	17
	Grassland	2	1	0	0	1
	Gully	0	0	15	0	3
	Channel bank	0	0	2	21	5
	Track	3	5	2	8	34
			Reclassification coefficient			67%

Figure S1 Linear discriminant analysis (LDA) showing the first and second discriminant functions (LD1 and LD2) of source reclassification using the selection of the composite fingerprints. Ellipses represent the 95%-confidence interval. The confusion matrix shows predicted (rows) and actual (column) number of samples with reclassification coefficient.
